# Synovial cyst of temporomandibular joint, a potential etiology for auriculotemporal neuralgia?

**DOI:** 10.1186/1129-2377-14-S1-P170

**Published:** 2013-02-21

**Authors:** H Ansari, CBE Robertson, I Garza

**Affiliations:** 1 Mayo Clinic, USA

## Introduction

Synovial cysts of the TemporoMandibularJoint (TMJ) are rare. To date, eleven case reports exist. AuriculoTemporal Neuralgia (ATN) is a distinct form of facial pain with no clear etiology. We report a case of synovial cysts of the TMJ with symptoms suggesting ATN.

## Purpose

To explore the relationship between synovial cysts of the TMJ and ATN.

## Method

Case report.

## Results

A 63-year-old female with left facial pain. This began intermittently and progressed to constant, dull, mild aching pain in the left temple and preauricular area with superimposed exacerbations of shooting and pulsatile pain. She described multiple paroxysmal jabbing, stabbing pains lasting 30 seconds each. These occurred in episodes lasting 1-15 minutes, five or six times/day, without clear triggers. Exam revealed tenderness anterior to tragus. Brain MRI/MRA were negative. Concern for temporal arteritis led to a prednisone trial and bilateral temporal artery biopsies, which were negative. She was then treated for presumed trigeminal neuralgia with carbamazepine and gabapentin, but could not tolerate the medications. She was on amitriptyline for fibromyalgia and we suggested she continue that treatment. Over next 18 months, she developed intermittent numbness/paresthesias in the preauricular area and ear canal. MRI of face revealed a 5 mm nonenhancing cyst in TMJ, anterior to left mandibular condylar process. Left arthrotomy and surgical pathology confirmed synovial cyst diagnosis.

## Conclusions

Articular branches of the auriculotemporal nerve innervate the TMJ joint, especially the lateral capsule. ATN is a distinct form of facial pain. Synovial cyst of the TMJ is a rare reported condition with varied symptoms based upon the size of the cyst. Our patient’s symptoms were highly suggestive of ATN, which had not been reported in the previous 11 cases. We recommend evaluation of the TMJ area in patients with idiopathic ATN.

## Conflict of interest

None.
